# Effects of GSH on Alcohol Metabolism and Hangover Improvement in Humans: A Randomized Double-Blind Placebo-Controlled Crossover Clinical Trial

**DOI:** 10.3390/nu16193262

**Published:** 2024-09-26

**Authors:** Gunju Song, Hyein Han, Seyoung Park, Soonok Sa, Wookyung Chung, Boo Yong Lee

**Affiliations:** 1 Department of Food Science and Biotechnology, College of Life Science, CHA University, Seongnam-si 13488, Republic of Korea; juhun022188@naver.com (G.S.); hyeinoo@naver.com (H.H.); 2 Food R&D, Samyang Corp., Seongnam-si 13488, Republic of Korea; seyoung.park@samyang.com (S.P.); soonok.sa@samyang.com (S.S.); wookyung.chung@samyang.com (W.C.)

**Keywords:** hangover, glutathione, yeast extract, acetaldehyde, alcohol metabolism

## Abstract

Background: The definition of alcohol hangovers refers to a combination of mental and physical side effects that occur after drinking. One of the ways that hangovers can be ameliorated is by promoting the rapid and effective elimination of acetaldehyde to alleviate the discomfort it causes. This study aimed to investigate the effects of GSH (yeast extract containing 50 mg of glutathione) on the hangover-relieving effect. Methods: A randomized double-blind placebo-controlled crossover clinical trial was conducted with 40 participants who reported experiencing hangover symptoms. Participants consumed alcohol at a rate of 0.78 g per kg body weight with 40% whiskey, adjusted according to their weight. Alcohol and acetaldehyde concentrations in serum were analyzed at 0, 0.25, 1, 2, 4, 6, and 15 h after alcohol consumption. Results: In the GSH group, the serum alcohol concentration decreased, although this change was not statistically significant. The serum acetaldehyde concentration was significantly lower in the GSH group in comparison to the placebo group (at 0.25, 1, 4, and 6 h (*p* < 0.01) and at 0.5, 2, and 15 h (*p* < 0.001) after alcohol consumption). However, there was no significant difference between the two groups on questionnaires such as the Acute Hangover Scale and the Alcohol Hangover Severity Scale. Conclusions: Overall, we consider the discovery that GSH lowered acetaldehyde concentration, a crucial factor in alcohol metabolism, to be more considerable. Therefore, GSH administration effectively reduces acetaldehyde levels in serum. This result suggests that this effect may contribute to the relief of hangover symptoms.

## 1. Introduction

Alcohol consumption is a widespread social activity around the world, but the resulting hangover leads to discomfort for many people [1,2]. Hangovers typically involve unpleasant physical and mental symptoms following alcohol consumption, such as dizziness, headaches, fatigue, and stomach pain [3,4]. These symptoms can affect daily activities, including work performance and driving. Therefore, managing hangovers is not only a medical concern but can also lead to a variety of social and economic issues [5,6]. Consequently, research focused on treating and preventing hangovers through various mechanisms is essential [7,8].

Upon the consumption of alcohol, approximately 90% of the alcohol absorbed via the stomach and intestines is transported to the liver for metabolic processing [9]. In the process of enzymatic oxidation, alcohol undergoes oxidation to form acetaldehyde by the enzyme alcohol dehydrogenase (ADH). This is followed by the conversion of acetaldehyde into acetate by aldehyde dehydrogenase (ALDH). The final products are then eliminated through urine excretion and exhalation as carbon dioxide [10,11]. However, constant and excessive alcohol consumption leads to the activation of the microsomal ethanol oxidation system instead of the dehydrogenase pathway. In this altered metabolic pathway, cytochrome P450 2E1 (CYP2E1) in the liver is activated instead of ADH, facilitating the breakdown of acetaldehyde [12]. The metabolism of alcohol by the enzyme CYP2E1 leads to the generation of excessive reactive oxygen species (ROS) and a decrease in glutathione levels within hepatocytes, which is an antioxidant crucial for scavenging ROS [13]. This reduction in glutathione levels is frequently associated with chronic alcohol consumption and liver disorders [14].

GSH (yeast extract containing glutathione) is a complex mixture containing amino acids, peptides, sugars, nucleic acids, lipids, B vitamins, and other components [15]. Glutathione, a peptide consisting of cysteine, glycine, and glutamate, serves as a powerful antioxidant and plays a crucial role in the detoxification of many toxic substances in the body [16,17,18]. Additionally, studies have shown that glutathione serves a crucial function in acetaldehyde tolerance as a direct scavenger of acetaldehyde [19,20]. Cysteinylglycine is considered a decomposition metabolite; research indicates that cysteinylglycine has the ability to undergo a reaction with acetaldehyde, resulting in the formation of 2-methylthiazolidine-4-carbonylglycine (MTCG) [14,21]. In consideration of the above, this human clinical trial aims to evaluate the effects and safety of GSH in improving hangover symptoms compared to a placebo.

## 2. Materials and Methods

All study designs and methodologies adhered to the Guidelines for Human Clinical Trials of Hangover Cure Claims established by the Ministry of Food and Drug Safety in Korea [22].

### 2.1. Subjects

This study was conducted with the approval granted by the Institutional Review Board (IRB) of CHA University, Bundang Medical Center (IRB No. CHAMC 2023-12-016). The study adhered to the fundamental ethical principles of the Declaration of Helsinki and the Korean Good Clinical Practice guidelines.

This clinical study participants were recruited from CHA University, Bundang Medical Center (Seongnam, Republic of Korea). The inclusion criteria were as follows: (1) healthy adults aged 19–40 years old, (2) a body mass index (BMI) between 18.5 to 25 kg kg/m^2^, (3) a history of consuming alcohol at least once a month and have consumed more than 1 bottle of soju (Korean distilled liquor), (4) have experienced a hangover after drinking alcohol, and (5) individuals who comprehended the detailed explanation of the study and voluntarily consented to participate and have given informed consent prior to the screening procedure. The exclusion criteria for the study were (1) participants with a clinically significant history of hypersensitivity, intolerance, or anaphylaxis to any of the main ingredients or other components of the Human Investigational Product (GSH). (2) Patients with a clinically significant medical history such as alcohol metabolism disorders, liver (such as severe hepatic impairment, gastrointestinal hemorrhage, gastrointestinal liver, hepatitis B and C carriers, alcoholic liver disease), kidney (such as severe renal impairment), digestive system (such as pancreatitis cholelithiasis), respiratory system, musculoskeletal system, endocrine system (diabetic ketoacidosis, diabetic coma and pre-coma, patients with type 1 diabetes), neuropsychiatric system, hematological and oncological system, cardiovascular system (heart failure, orthostatic hypotension, hypertension), gout, active tuberculosis were excluded. (3) Participants were excluded if they had a history of gastrointestinal disease (e.g., Crohn’s disease, ulcerative disease) or surgery (but not appendectomy, hernia surgery, endoscopic polyp surgery, hemorrhoids, dentistry, or fistula surgery) that could affect the absorption of the Human Investigational Product (GSH). (4) Patients with alcohol use disorder or alcohol dependence at screening and (5) those who consumed excessive alcohol (>210 g/week) within 1 week or were unable to stop alcohol consumption from 48 h prior to the administration of each period until discharge from the hospital were also excluded. (6) Participants were excluded if they had abnormal Alanine Transaminase (ALT), Aspartate Transaminase (AST), or Total bilirubin (2.5 times the standard upper limit) levels and Chronic Kidney Disease Epidemiology Collaboration (CKD-EPI) of less than 60 mL/min/1.73 m^2^ were excluded. (7) In addition to this, individuals were excluded if an abnormal result on any of the screening tests was deemed clinically significant. (8) Participants who had received an investigational drug or investigational food product in another clinical trial or human application within 30 days of screening were excluded. (9) Participants who were taking ongoing antabuse medications or dietary supplements that may affect alcohol metabolism were excluded, as were those taking ongoing medications that may cause bleeding events, such as warfarin, clopidogrel, aspirin, and NSAIDs. (10) Also excluded were those who had received liver function-enhancing medications, antibiotics, choleretic agents, antioxidants, or dietary supplements within 14 days of the first dose of the investigational product, or who did not agree to abstain from such medications from 14 days to the end of the study, (11) and those who had received drugs that induce or inhibit drug metabolizing enzymes, such as barbiturates, within 30 days of the first dose of the investigational product, or who did not agree to abstain from such drugs from 30 days to the end of the study. (12) Pregnant or lactating women, or those with a positive pregnancy test, were not eligible to participate in this study. (13) In addition to the above, those deemed unsuitable by the investigator for participation in this human application study were also excluded.

### 2.2. Study Design

This clinical trial was designed as a randomized double-blind crossover placebo-controlled trial and it adhered to the Guidelines for Human Clinical Trials of Hangover Cure Claims established by the Ministry of Food and Drug Safety in Korea [22]. Volunteers were required to provide written informed consent, and 40 participants were selected after screening tests were conducted to determine the inclusion of participants in the study. Selected participants were randomly assigned to either Sequence A or Sequence B on Day 0 and assigned a subject identification number. Both the participants and the investigators remained unaware of the intervention assignments until the conclusion of the study. The randomization table and associated lists were maintained separately by the randomization table manager. The sample size necessary to achieve 80% statistical power at a 5% significance level was determined to be 30 participants per group, which is considered suitable for clinical research. To account for a projected dropout rate of 25%, it was estimated that a total of 40 participants would be needed to determine effectiveness. A one-week interval was implemented between Sequence 1 and Sequence 2 to remove any carry-over effects.

### 2.3. Interventions and the Alcohol Challenge Test

The GSH (yeast extract containing glutathione) component analysis data are presented in (Table 1), indicating that the glutathione content in GSH was 21.25% (wet basis). The glutathione dose of GSH used in this study (50 mg) is based on preliminary animal studies and the content of commercially available products. The GSH used in this study was obtained from Samyang Corporation, Republic of Korea, and is suitable for the Korean Food Additives Code published by the Korean Food and Drug Administration [23]. And so, we used GSH (Yeast extract containing 50 mg of glutathione/235.3 mg GSH) capsules (235.3 mg/capsule) or placebo capsules (cellulose, 235.3 mg) for oral administration. Each sample was identical in appearance. All participants received an identical meal (standard diet) prior to the trial and administrated either the placebo or GSH capsules with 150 mL water at 2 h after meal ingestion. Thirty minutes after ingesting each capsule, participants consumed whiskey with 40% alcohol content (alcohol content 0.78 g/kg bw) and shrimp snacks (20 each, 60 kcal) within 30 min. Drinking water was prohibited from 2 h prior to the administration of each capsule, except for drinking water consumed at dinner (100 mL), and 150 mL is allowed during the 30 min of alcohol consumption and 150 mL at 2 and 4 h after the placebo or GSH capsules administered. Participants were accommodated overnight at the clinical research center. Upon awakening the following morning, participants completed a hangover symptom questionnaire 12 h after consuming alcohol, in addition to undergoing a physical examination, vital signs assessment, and clinical laboratory tests. This protocol is in accordance with the Guidelines for Human Clinical Trials of Hangover Cure Claims established by the Ministry of Food and Drug Safety in Korea [22].

### 2.4. Assessment of Hangover Severity

The Acute Hangover Scale (AHS) includes 9 items: ‘hangover’, ‘thirsty’, ‘tired’, ‘headache’, ‘dizziness/faintness’, ‘loss of appetite’, ‘stomachache’, ‘nausea’, and ‘heart racing’. Each item is scored on a scale from 0 to 7, with the following anchors: ‘none’ corresponding to a score of 0, ‘mild’ to a score of 1, ‘moderate’ to a score of 4, and ‘incapacitating’ to a score of 7. Data from AHS assessments were collected at 1, 4, and 12 h after alcohol consumption.

The Alcohol Hangover Severity Scale (AHSS) includes 12 items: ‘fatigue’, ‘apathy (lack of interest/concern)’, ‘concentration problems’, ‘clumsiness’, ‘confusion’, ‘thirst’, ‘sweating’, ‘shivering’, ‘stomach pain’, ‘nausea’, ‘dizziness’, and ‘heart pounding’. Each item is rated on a scale from 0 to 10 (0 = absence of symptom, 10 = extreme symptom). The data from AHSS assessments were collected 12 h after alcohol consumption. The questionnaire was selected and measured in accordance with the Guidelines for Human Clinical Trials of Hangover Cure Claims established by the Ministry of Food and Drug Safety in Korea [22].

### 2.5. Safety

Vital signs such as blood pressure (systolic and diastolic blood pressure), body temperature, pulse rate, and clinical laboratory tests (hematology, biochemical, and urine tests) were performed on the participants at each visit. Furthermore, throughout the duration of the study, any adverse events were verified through interviews or questionnaires with the participants.

### 2.6. Alcohol and Acetaldehyde Analysis in Serum

Alcohol and acetaldehyde concentrations were measured in accordance with the Guidelines for Human Clinical Trials of Hangover Cure Claims established by the Ministry of Food and Drug Safety in Korea [22]. In total, 5 mL of blood was collected from participants to measure alcohol and acetaldehyde concentrations. Blood samples were collected in the vein at various time intervals, specifically, at 0, 0.25, 0.5, 1, 2, 4, 6, and 15 h after alcohol consumption. A saline-locked angiocatheter was inserted into the participant’s arm, allowing for the collection of 5 mL of blood at each designated time point. The samples were then placed in a VACUETTE tube and stored at a temperature of 4 °C for a duration of 30 min. The whole blood that was collected was centrifuged at 2500× *g* at 4 °C for 10 min. After that, the serum was transferred to a screw cap tube and kept at −70 °C for storage. Then, samples were transported to the analytical laboratory located at CHA University, Department of Food Science and Biotechnology (Seongnam, Republic of Korea). The alcohol concentration in serum was measured using the ethanol assay kit (Abcam, ab65343, Cambridge, UK). The protocol involved mixing serum with the EtOH assay buffer, followed by the addition of 50 µL of the reaction mixture. After incubating at room temperature in the dark for 60 min, the absorbance was measured at 570 nm. The limit of detection (LOD) for this kit is 0.1 ppm. Additionally, the acetaldehyde concentration in serum was measured using the aldehyde assay kit (Abcam, ab219923, Cambridge, UK). The protocol required mixing serum with the assay buffer and then adding 50 µL of the reaction mixture. After incubating at room temperature in the dark for 30 min, the absorbance was measured at 620 nm. The LOD for this kit is 3 μM.

### 2.7. Statistical Analysis

Statistical analyses were performed using IBM^®^ SPSS^®^ Statistics software (version 27.0; Armonk, NY, USA). The data collected from this clinical trial were analyzed by calculating the means standard deviations (SD), along with appropriate descriptive statistics, and the significance of the differences was two-tailed at *p* < 0.05. A paired *t*-test was used for comparisons within groups. An independent *t*-test was used to compare the means of two independent groups. Categorical variables were compared using the chi-squared test or Fisher’s exact test. Alcohol and acetaldehyde pharmacokinetic parameters were analyzed by PhoenixWinNonlin^®^ software (version 8.4; Certara, Princeton, NJ, USA).

## 3. Results

### 3.1. Enrollment

**Figure 1:**
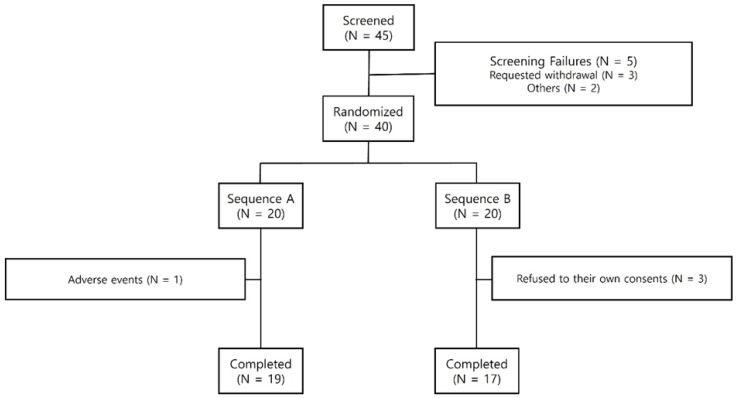
Subject disposition.

A total of 45 participants were enrolled in the study following the exclusion of 5 individuals during the screening tests, which included questionnaires, physical examinations, and laboratory tests. This resulted in 20 participants assigned to Sequence A and 20 participants assigned to Sequence B. The enrollment of participants occurred within four weeks following the screening tests. Additionally, prior to the trial, informed consent was obtained from all participants. During the trial, four participants (one in Sequence A and three in Sequence B) were excluded from the final analysis (Figure 1). The Safety Analysis was conducted on 40 participants who received treatment at least once. The Pharmacodynamic Analysis was conducted on 36 participants (19 participants in Sequence A and 17 participants in Sequence B).

### 3.2. General Participant Characteristics

Table 2 presents a comparative analysis of the demographic information of the participants. A comprehensive examination of all characteristics, including demographic data, was conducted between Sequence A and Sequence B to identify any significant differences. The analysis showed that there were no statistically significant differences between the groups regarding sex, height, age, fertility (in women only), exercise, smoking, or body mass index (BMI). These findings suggest that the randomization process was successful, thereby enhancing the reliability and validity of the study’s results. Furthermore, they indicate that these demographic factors did not serve as confounding variables in this study.

### 3.3. Biochemical Parameters

12 hours after alcohol consumption, vital signs (SBP, DBP, and PR) and laboratory tests (AST, ALT, Total bilirubin, ALP, γ-GTP, BUN, Creatinine, Glucose, and Uric acid) were measured. No statistically significant differences in vital signs or laboratory tests were observed when comparing the GSH group and the placebo group (Table 3). This suggests that GSH treatment is safe for humans.

### 3.4. Change in Alcohol and Acetaldehyde Levels

**Figure 2:**
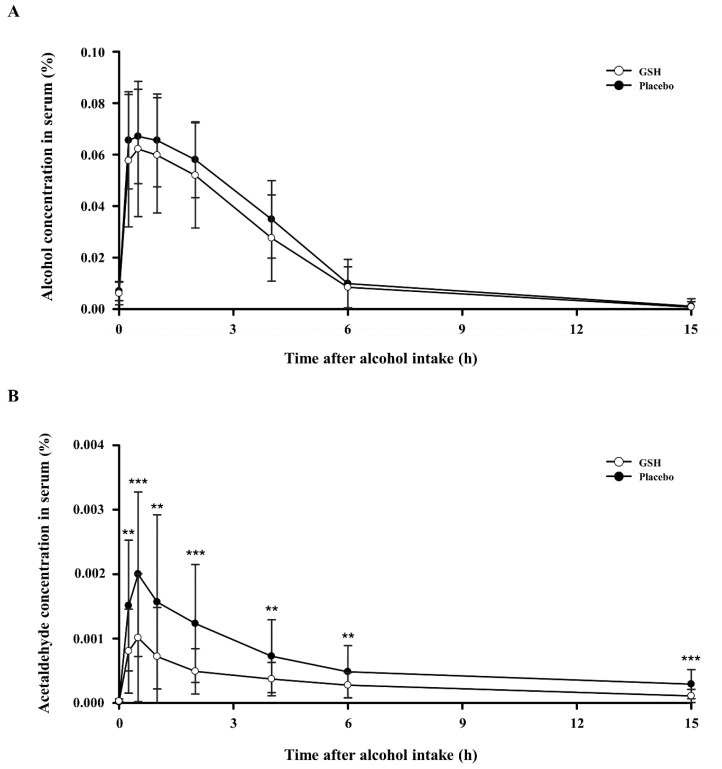
Effects of GSH on serum concentrations of alcohol and acetaldehyde after alcohol consumption: (**A**) serum alcohol levels and (**B**) serum acetaldehyde levels. Compared between groups; *p*-value for the Paired *t*-test. ** *p* < 0.01, *** *p* < 0.001.

We measured alcohol and acetaldehyde concentrations in the serum to confirm the hangover improvement effect of GSH. Figure 2 shows the findings related to the alterations in concentrations of alcohol and acetaldehyde in serum from 0 to 15 h after alcohol consumption. The serum alcohol concentration was lower in the GSH group compared to the placebo group, but the difference was not statistically significant (Figure 2). However, the acetaldehyde concentration in serum was significantly lower in the GSH group compared to the placebo group at all time points, indicating that GSH treatment effectively reduced acetaldehyde, which is a direct cause of hangovers.

Next, we measured the pharmacokinetic parameters of alcohol and acetaldehyde. Table 4 presents the maximum serum concentrations of alcohol and acetaldehyde (C_max_), the time required to reach these peak concentrations (T_max_), and the area under the curve (AUC) from 0 to 15 h after alcohol consumption. The serum alcohol levels showed a reduced C_max_ and AUC in the GSH group when compared to the control group, although this difference was not statistically significant. Conversely, the serum acetaldehyde levels showed significantly lower C_max_ and AUC in the GSH group compared to the control group, with a statistically significant difference. T_max_ for both alcohol and acetaldehyde were the same for the GHS-treated and control groups.

### 3.5. Survey of Hangover Symptoms

The AHS survey is conducted at 1, 2, and 12 h after alcohol consumption, and the AHSS survey is conducted at 12 h after alcohol consumption to compare the GSH and control groups. However, no significant differences were observed in the overall AHS and AHSS results. Next, we analyzed the AHS and AHSS survey data by time (1, 2, and 12 h), age, and gender. Although the overall data were not statistically significant, some items were significant at the 90% level for females. Among the nine items in the AHS results for females, the data showed that ‘Hangover’ and ‘Tired’ improved 1 h after alcohol consumption, and ‘Thirst’ improved 4 h after alcohol consumption. Additionally, among the 12 items in the AHSS results for females, the data showed that ‘Thirst’ improved 12 h after alcohol consumption. However, these results are based on some items only in females. Therefore, we conclude that there is no significant difference in overall hangover symptoms.

## 4. Discussion

This study involved clinical trials with GSH (yeast extract containing glutathione) that were conducted to evaluate its effectiveness in facilitating alcohol metabolism in the human body and alleviating symptoms associated with hangovers. Glutathione in yeast extract is a powerful antioxidant in the body, involved in a variety of metabolic processes through redox reactions [24,25,26]. Pollock, which is known to be particularly high in glutathione, has been commonly used in folk medicine since ancient times to relieve hangovers.

Alcohol and its metabolite acetaldehyde are considered toxic and harmful to humans. Among them, the one that has a more direct effect on hangovers is acetaldehyde. Acetaldehyde is a strong electrophilic compound produced endogenously as the initial intermediate in oxidative ethanol metabolism. It exhibits high reactivity toward nucleophiles in vivo, making it toxic [27,28,29]. The accumulation of acetaldehyde in the body, when not converted to acetate, leads to hangover symptoms. In this study, we assessed serum alcohol and acetaldehyde levels to evaluate the potential hangover-relieving effect of GSH. The alcohol concentration in serum showed a decrease in the GSH group compared to the placebo group, but the difference was not statistically significant, and the AUC and C_max_ were not statistically different. In addition, the acetaldehyde concentration in serum significantly decreased in the GSH group compared to the control group, with a statistical reduction in AUC and C_max_. These findings suggest that GSH can effectively lower alcohol absorption and promote acetaldehyde elimination during alcohol metabolism. Several studies have demonstrated that cysteinylglycine, the primary metabolite in glutathione breakdown by gamma-glutamyltranspeptidase, exhibited rapid and equimolar reactivity with acetaldehyde, comparable to acetaldehyde’s reaction [14,21,30,31]. This suggests that the glutathione present in GSH helps eliminate acetaldehyde.

The AHS survey is conducted at 1, 2, and 12 h after alcohol consumption, and the AHSS survey is conducted at 12 h after alcohol consumption, and significant differences were found between the GSH and placebo groups in either survey. However, we consider acetaldehyde levels to be a more objective biochemical marker than the questionnaire results. This is because the AHS and AHSS surveys rely on subjective assessments that reflect participants’ personal experiences, which can have limitations. A limitation of this study is that while acetaldehyde levels in serum have been objectively measured, the results of the hangover symptom questionnaire are highly subjective, and their standard deviations are very big, so there is no significant difference. This likely may not have fully captured the true physiological effects of GSH. While the reduction in acetaldehyde is significant, the lack of a clear objective connection between this reduction and the alleviation of hangover symptoms restricts the ability to directly assert that GSH alleviates hangover symptoms.

There is another study that showed a significant reduction in acetaldehyde, a toxic byproduct of alcohol metabolism that causes hangovers [32]. This study indicated a dose-dependent decrease in acetaldehyde levels by enhancing alcohol metabolism, as well as variations in the questionnaire regarding hangover symptoms. However, the sample size was relatively small compared to this study, which may limit the generalizability of these findings.

In conclusion, in this study, GSH (yeast extract containing glutathione) effectively reduces acetaldehyde levels in serum. This result suggests that this effect may contribute to the relief of hangover symptoms. Additional studies are needed to explore the mechanism of GSH in relieving hangover symptoms.

## 5. Conclusions

The present study shows that the administration of GSH (yeast extract containing glutathione) effectively reduces acetaldehyde levels in serum. This result suggests that this effect may contribute to the relief of hangover symptoms. These findings suggest that GSH may be an effective supplement for mitigating hangovers.

## Figures and Tables

**Table 1:** The analysis data of the GSH (yeast extract containing glutathione).

Components	Contents (%, wb)
Moisture	4.1
Total carbohydrate	2.13
Total fat	0.07
Protein	72.01
Ash	21.69
Total Contents	100
Total Glutathione	21.25 (%, wb)
Arsenic (not more than 2 ppm)	<2
Heavy metals (not more than 20 ppm)	<20
Total plate count (not more than 3000/g)	<5
Mold and Yeast (not more than 100/g)	<5
*Coliform bacteria*	Negative
*Salmonella*	Negative
*Staphylococcus aureus*	Negative
*Bacillus cereus* (not more then 100/g)	<5
*E. coli*	Negative

**Table 2:** Demographic characteristics.

		Sequence		Total	
		Sequence A	Sequence B		*p*-Value
		(n = 19)	(n = 17)	(n = 36)	
Age (Years)	Mean ± SD	29.63 ± 4.70	30.06 ± 5.72	29.83 ± 5.14	0.8073 ^†^
	Min, Max	21.00, 38.00	21.00, 40.00	21.00, 40.00	
Height (cm)	Mean ± SD	129.63 ± 4.70	130.06 ± 5.72	171.21 ± 6.67	0.5673 ^†^
	Min, Max				
Weight (kg)	Mean ± SD	65.37 ± 6.05	65.11 ± 9.33	65.24 ± 7.66	0.9199 ^†^
	Min, Max	55.50, 74.90	48.70, 78.00	48.70, 78.00	
BMI (kg/m^2^)	Mean ± SD	22.46 ± 1.89	21.95 ± 2.15	22.22 ± 2.00	0.4483 ^†^
	Min, Max	19.30, 24.70	18.70, 24.80	18.70, 24.80	
Sex n (%)	Male	16 (84.21)	13 (76.47)	29 (80.56)	0.4335 ^‡^
	Female	3 (15.79)	4 (23.53)	7 (19.44)	
Fertility n (%)	Yes	3 (15.79)	4 (23.53)	7 (19.44)	-
	No	0 (0.00)	0 (0.00)	0 (0.00)	
Exercise n (%)	No	6 (31.58)	6 (35.29)	12 (33.33)	0.2598 ^¶^
	1–2 per week	3 (15.79)	7 (41.18)	10 (27.78)	
	3–4 per week	8 (42.11)	3 (17.65)	11 (30.56)	
	5–6 per week	1 (5.26)	1 (5.88)	2 (5.56)	
	Every day	1 (5.26)	0 (0.00)	1 (2.78)	
Smoking n (%)	Current smoker	5 (26.32)	5 (29.41)	10 (27.78)	0.0764 ^¶^
	Ex-smoker	0 (0.00)	3 (17.65)	3 (8.33)	
	Non-smoker	14 (73.68)	9 (52.94)	23 (63.89)	

Values are presented as mean ± standard deviation or number (%). ^†^ Independent *t*-test, ^‡^ Fisher’s exact test, ^¶^ Chi-square test.

**Table 3:** Vital signs and laboratory tests at 12 h after alcohol consumption.

	Treatment		*p*-Value ^†^
	GSH	Placebo	
	(n = 36)	(n = 36)	
Vital signs			
SBP (mmHg)	119.58 ± 9.12	118.94 ± 10.93	0.6920
DBP (mmHg)	70.39 ± 8.69	68.33 ± 8.55	0.0934
PR (BPM)	65.75 ± 8.60	65.53 ± 7.66	0.8503
Laboratory tests			
AST (IU/L)	20.47 ± 25.40	16.25 ± 5.30	0.3058
ALT (IU/L)	16.39 ± 11.76	15.53 ± 9.43	0.6837
Total bilirubin (mg/dL)	0.75 ± 0.23	0.71 ± 0.24	0.2557
ALP (IU/L)	60.53 ± 18.72	60.75 ± 19.55	0.7893
γ-GTP (U/L)	20.72 ± 19.42	20.58 ± 16.57	0.8748
BUN (mg/dL)	12.00 ± 2.12	11.73 ± 2.19	0.4489
Creatinine (mg/dL)	0.80 ± 0.11	0.80 ± 0.14	0.6722
Glucose (mg/dL)	87.94 ± 5.68	88.83 ± 5.68	0.2721
Uric acid (mg/dL)	5.65 ± 1.48	5.55 ± 1.38	0.1823

Data are expressed as mean ± standard deviation. Abbreviations: SBP, systolic blood pressure; DBP, diastolic blood pressure; PR, pulse rate; AST, aspartate aminotransferase; ALT, alanine aminotransferase; ALP, Alkaline Phosphatase; γ-GTP, γ-glutamyl transferase; BUN, blood urea nitrogen. ^†^ Paired *t*-test.

**Table 4:** Alcohol and acetaldehyde pharmacokinetic parameters.

		Treatment		GMR (90% CI)	
		GSH	Placebo		*p*-Value ^†^
		(n = 36)	(n = 36)		
Alcohol	C_max_ (%^-^10^−2^)	6.53 ± 2.69	7.09 ± 1.93	0.8904 (0.7627–1.0395)	0.3949
	AUC_last_ (%^-^10^−2^·h)	26.62 ± 12.61	30.24 ± 11.95	0.8226 (0.6714–1.0077)	0.2136
	T_max_ (h)	0.50 [0.25–2.00]	0.50 [0.25–2.00]	-	-
Acetaldehyde	C_max_ (%^-^10^−4^)	10.77 ± 9.34	22.70 ± 14.21	0.4764 (0.3668–0.6187)	0.0002
	AUC_last_ (%^-^10^−4^·h)	48.80 ± 36.20	99.54 ± 62.05	0.5158 (0.3815–0.6975)	0.0003
	T_max_ (h)	0.50 [0.25–4.00]	0.50 [0.25–4.00]	-	-

Data are expressed as mean ± standard deviation, except for T_max_, which is expressed as median [minimum–maximum]. Abbreviations: C_max_, maximum serum concentration; AUC_last_, AUC from 0 to last measurable time point; T_max_, time to reach C_max_. ^†^ Paired *t*-test.

## Data Availability

The data from the manuscript are information collected from human participants, so they are not available due to confidentiality reasons.
